# Viral phylogeny in court: the unusual case of the Valencian anesthetist

**DOI:** 10.1186/1741-7007-11-83

**Published:** 2013-07-19

**Authors:** Anne-Mieke Vandamme, Oliver G Pybus

**Affiliations:** 1 Laboratory for Clinical and Epidemiological Virology, Rega Institute for Medical Research, Department of Microbiology and Immunology, University of Leuven, Leuven, Belgium; 2 Centro de Malária e outras Doenças Tropicais and Unidade de Microbiologia, Instituto de Higiene e Medicina Tropical, Universidade Nova de Lisboa, Lisbon, Portugal; 3 Department of Zoology, University of Oxford, South Parks Road, Oxford, OX1 3PS, UK

## Abstract

A large and complex outbreak of hepatitis C virus in Valencia, Spain that began 25 years ago led to the prosecution and conviction of an anesthetist who was accused of infecting hundreds of his patients. Evolutionary analyses of viral gene sequences were presented as evidence in the trial, and these are now described in detail by González-Candelas and colleagues in a paper published in *BMC Biology*. Their study illustrates the challenges and opportunities that arise from the use of phylogenetic inference in criminal trials concerning virus transmission.

See research article: http://www.biomedcentral.com/1741-7007/11/76

## 

During the past 20 years molecular phylogenetics has become increasingly popular as a tool for the forensic investigation of viral transmission, where it is used to infer the ancestral relationships of infections from sampled viral genome sequences. It has been applied most commonly to cases involving the transmission of the fast-evolving human immunodeficiency virus (HIV). Like HIV, hepatitis C virus (HCV) is a globally prevalent, blood-borne and rapidly evolving virus, yet it is unusual for its transmission to be evaluated scientifically in a criminal court. In a paper in *BMC Biology*, González-Candelas *et al*. [1] describe the application of viral phylogenetics to an exceptionally large and complex outbreak of HCV in Valencia, Spain, that began around 25 years ago and occurred over a period of several years. Their analysis contributed to the successful prosecution of an anesthetist who, it is thought, infected hundreds by sharing syringes containing analgesics with patients shortly after surgical operations. The case illustrates the theoretical and practical challenges facing those who provide expert phylogenetic testimony in court.

One of the difficulties in analyzing the transmission of very fast-evolving viruses such as HIV and HCV is that the genomes of strains sampled from individuals who are known without doubt to have infected each other are rarely identical. They are, however, likely to be genetically more similar than those taken from epidemiologically unlinked individuals; hence, viral phylogenies can help to evaluate proposed transmission scenarios. Phylogenetics was first used for forensic purposes in 1992 in the ‘Florida dentist case’ to investigate the possible transmission of HIV from a dentist to seven of his patients [2], although in that instance the evidence was used only to settle out of court. A few years later HIV transmission through rape was investigated in Sweden using phylogenetic methods and the analysis, together with other evidence, condemned the accused in court. The subsequent publication of that study set the stage for the use of viral phylogenies as evidence in court [3,4].

Gonzalez-Candelas *et al*. [1] explain in their paper that they were asked by the Court to address several questions, including whether the defendant was in fact the source of the Valencian outbreak; which of 332 patients he was suspected of infecting he did in fact infect; and when each had been infected. As is common in criminal cases involving viral transmission, the phylogenetic analysis presented by González-Candelas *et al*. [1] was not used to prove guilt by itself, but rather served to support other lines of evidence. The estimation and interpretation of viral phylogenies are inherently uncertain and therefore they should be used in court very differently to DNA fingerprints [5], which can uniquely identify individuals with a very high degree of probability. First, because HIV and HCV evolve rapidly, viral sequences within one individual can be highly diverse and change through time. Consequently, the branching events in a viral phylogeny do not always correspond to the transmission events among sampled infections. Second, sequences collected for a given investigation may not represent all the individuals belonging to the transmission chain under consideration. This means that infections belonging to the same local epidemic can cluster together phylogenetically even if there has been no direct transmission between the individuals sampled, because of transmission from one or more unsampled individuals [5]. In short, the existence alone of genetic similarity or phylogenetic linkage among viruses cannot show beyond doubt that direct transmission occurred between two individuals.

One technique used to reduce the chance clustering of viral sequences is the inclusion of ‘local controls’ - that is, viral sequences from infected individuals in the same location who are not believed to be part of the outbreak under investigation. But even when many local control infections are used, the observation of strong phylogenetic clustering of sequences from the defendant and complainant does not definitively show that one infected the other, because the possibility remains that the complainant was infected by an individual not included in the analysis [5-7].

The inclusion of appropriate local control sequences does, however, increase the likelihood of observing phylogenetic separation of sequences from the defendant and complainant in instances where direct transmission did not occur, and such separation can provide reasonable doubt of the defendant’s guilt. The outbreak investigated by González-Candelas *et al*. [1] was unusual in its exceptional size and duration and consequently the ratio of the number of local controls to possible complainants was unavoidably lower than in other investigations, which reduces the strength of the phylogenetic conclusions that can be drawn. Further, the complexity and size of the Valencian case increases the chance that complainants may belong to the same network of transmission without having been infected directly by the defendant, and it is conceivable that some putative recipients had risk factors for HCV infection other than surgery with the accused anesthetist. Similarly, it is possible that the accused was infected with HCV from one of his patients before subsequently transmitting the virus to many others.

All of the above highlights the importance of evidence other than phylogenetic analysis when building a case for direct transmission - for example, evidence gained through medical records and interviews. Contact tracing is a key investigatory tool but not without its own limitations, as it is dependent on the testimony of the complainants who must be able or willing to disclose all behaviors that could have led to transmission, some of which may be illegal or socially stigmatized (for example, illicit drug injection). When available, other laboratory data such as serology may help to establish a time window during which infection occurred. When the windows of infection for two individuals do not overlap then the person found to have been infected later clearly cannot have caused the earlier infection.

Phylogenetic evidence regarding transmission can often be improved by sequencing a large number of virus genomes from each infected individual. In their study González-Candelas *et al*. [1] obtained 134 sub-genomic sequences from the defendant and an average of 10 from each putative recipient. It has been argued previously, in the context of HIV transmission [8], that such data can help to establish the direction and source of transmission when the complainant’s viruses are nested within the genetic variability found in the defendant (in phylogenetic jargon, the latter are 'paraphyletic' with respect to the former). However, demonstrating direction of transmission is not identical to demonstrating direct transmission from one to another. It is fair to say that experts in viral phylogenetics differ in their views on the weight that can be placed on such nesting. Some think it can be used as useful and credible information for the prosecution, whilst others argue that it is uncertain and should be used only conservatively, to allow the defense to establish doubt as to a proposed source or direction of transmission. In the Valencian case paraphyly was found for some but not all proposed recipients. As González-Candelas *et al*. note [1], special difficulties arise in applying the principle of paraphyly to HCV because it is difficult to assess the true diversity of the viral population within an infected individual. Since HCV replicates mainly in the liver, it is possible that any single blood sample captures only a subset of within-host viral diversity [9], and in the case of the anesthetist only one blood sample was available (the defendant had the right under Spanish Law to refuse to give more).

An additional problem that may apply in populations at high risk of HCV infection is that individuals may clear infection only to be re-infected shortly thereafter with a closely related viral strain, which may preclude the use of paraphyletic clustering to support or exclude a particular direction of transmission.

A further notable aspect of the results presented by González-Candelas *et al*. [1] is an attempt to estimate from genetic data alone a possible time of infection for each putative victim (see also [10]). To tackle this question, they used evolutionary molecular clock models, which convert the amount of genetic change among strains into an estimate of their time of divergence. The molecular clock estimates of infection date were then correlated with those proposed by the prosecution (that is, dates when the complainant underwent surgery attended by the defendant), with variable success: the confidence limits of the molecular clock estimates included the prosecution’s proposed date in two out of every three cases. To our knowledge this is the first use of such methods as evidence in a criminal trial. Molecular clock models play an important role in modern molecular epidemiology [11] and have been used to establish a timescale of transmission for HIV and HCV outbreaks (for example, [12]). However, the confidence limits associated with molecular clock estimates can sometimes be very wide and the meaning of this uncertainty may be difficult to communicate to a jury.

Clearly, many factors can affect the accuracy and reliability of both paraphyletic clustering and of molecular clock divergence times. These include sample size, the temporal and anatomical distribution of sampling, the viral genome region sequenced, whether or not super- or re-infection occurred, and the precise evolutionary models and algorithms employed. The use of these two techniques in criminal investigations needs further validation using documented transmission histories. Such validation studies have previously proved valuable in exploring both the power and the limitations of phylogenies to reliably reconstruct known chains of transmission using conventional sequencing techniques (for example, [6,7,13,14]).

Although the analysis of the Valencian HCV outbreak illustrates some of the challenges inherent in the phylogenetic analysis of HCV transmission in criminal investigations, many aspects of the case are atypical, including the very large number of possible recipients of transmission, the length of time over which the infections occurred, and the lack of recipient-donor paraphyly in some instances. Some technical aspects of the investigation, such as the use of phylogenetic hypothesis tests and molecular clock analyses will likely spur debate among experts in viral forensics. Finally, it should be noted that the investigation was undertaken some time ago, before sequencing of whole HCV genomes from large cohorts became affordable and using computer hardware less powerful than that available today. Since then, highly parallel ‘deep’ sequencing approaches to within-host viral genetic diversity have become more common; whether such sequencing technologies might be applied successfully to forensic viral transmission cases remains to be seen. But perhaps the most intractable problem facing phylogenetic experts in court is neither scientific nor statistical, but instead arises from the perception of genetic evidence held by lay jurors. Accustomed by exposure to popular forensic television dramas, and to media reports of convictions obtained through DNA fingerprinting, there is a risk that jurors may see phylogenetic evidence in the same light, and fail to comprehend or fully engage with its complexities and inherent shortcomings.

### Abbreviations

HCV: Hepatitis C viru; HIV: Human immunodeficiency virus.
